# Childhood Socioeconomic Status and Depressive Symptoms of Young Adults: Mediating Role of Childhood Trauma

**DOI:** 10.3389/fpsyt.2021.706559

**Published:** 2021-11-30

**Authors:** Caiyan Yang, Peiyi Chen, Junyi Xie, Yongtong He, You Wang, Xueling Yang

**Affiliations:** ^1^Department of Psychology, School of Public Health, Southern Medical University, Guangzhou, China; ^2^Department of Psychiatry, Zhujiang Hospital, Southern Medical University, Gaungzhou, China; ^3^Guangdong Provincial Key Laboratory of Tropical Diseases, Southern Medical University, Guangzhou, China

**Keywords:** socioeconomic status, depressive symptoms, childhood trauma, young adults, China

## Abstract

**Background:** Studies have shown that low childhood socioeconomic status (SES) is associated with a high prevalence of depressive symptoms. Childhood trauma, as a potential consequence of low SES, may play an important part, but the mediation effect of childhood trauma remains to be elucidated.

**Methods:** A cross-sectional survey was conducted among 1,807 university students. The MacArthur Scale of Subjective Social Economic Status—Youth Version, Childhood Trauma Questionnaire, and Beck Depression Inventory were used to measure childhood SES, childhood trauma, and current depressive symptoms, respectively. A structural equation model (SEM) was employed to demonstrate the mediating role of childhood trauma on the association between childhood SES and depressive symptoms.

**Results:** The SEM demonstrated that childhood SES had significant indirect effects upon depressive symptoms *via* childhood trauma. Childhood trauma accounted for 89.3% of the total effect, indicating a profound mediation effect.

**Conclusions:** The effect of childhood SES on the depressive symptoms of young adults was mediated by childhood trauma, which emphasizes the importance of early prevention and intervention of child neglect/abuse.

## Introduction

Studies have demonstrated that socioeconomic status (SES) is associated with the risk of major depressive disorders. One meta-analysis concluded that low SES was associated with an increased risk of depression worldwide [odds ratio (OR) = 1.81, 95% confidence interval (CI) 1.6–2.1] (1).

Consistently, rural residents in China have shown a higher incidence of depression, compared with individuals living in urban areas, with an inverse relationship between family income and depression observed (2). The risk of depression for individuals living in rural areas and with low SES has been shown to be 1.5- and 2.3-fold higher, respectively, than that for individuals living in urban areas (OR 1.5, 95% CI 1.4–1.7; OR 2.3, 95% CI 2.1–2.4) (3).

Theories based on social causation and health selection have been proposed to explain the relationship between SES and mental disorders (4). Social causation theory suggests that higher levels of stress and adversity caused by low SES account for the observed elevation in the risk of psychological disorders. Health selection theory assumes that mental disturbances interfere with upward mobility or result in downward mobility, which, in turn, influences SES in individuals with mental disorders. While previous research favors social causation theory, evidence from China examining these theories is scarce. Furthermore, psychosocial mechanisms underlying the effect of SES on depression merit additional exploration.

Studies have shown that the effects of SES on mental health vary by the age of experience. For example, childhood SES (which is determined by family economic status) has been found to play a profound role in self-rated health, relative to adulthood SES, and mental disorders are primarily influenced by early-life SES (5). Evidence regarding the amelioration of these effects by SES improvement later in life are mixed, with some research showing that improvement of SES in adulthood may not reduce the adverse effect of childhood SES on adult depression (6, 7) and other research showing that the negative influence of childhood SES on adult depression may be buffered by a rise of SES in adulthood, with improvement of SES decreasing the risk of adult depression (8). However, if there is a failure in improvement of adulthood economic status, then the probability of developing depression may be increased (9). These disparate results may be due to a mixture of the effects of childhood SES and adulthood economic status in adult cohorts that were examined in previous studies. As the current study focuses on young adults, it should be noted that, in China, >90% of university students are dependent upon their family members for financial support (10, 11). The first and second year undergraduate students are selected in this study because their financial resources are mainly from their families as mentioned before, while college students in their third year and above usually start to do part-time jobs and begin to have other financial sources (12). If we choose the third year and above undergraduate college students, their other financial sources will be confounding factors of our research. As a result, we choose the first and second year students to reduce the influence of other financial sources.

Childhood SES has also been shown to be closely related to physical trauma, emotional trauma, and/or sexual abuse during childhood (13). Cross-sectional research has shown that Childhood Trauma Questionnaire (CTQ) scores in high-income countries are relatively low (14), whereas childhood trauma occurs more frequently in low-SES areas (13). A United Nations International Children's Emergency Fund report found a need for national-level data on violence against children in China (15). Similar to SES, childhood trauma has also been shown to be an important risk factor for depression development (16–20). For example, one study exploring the predictors of depressive symptoms in Chinese college students found that a greater childhood abuse was associated with more severe depressive symptoms (OR 1.05, 95% CI 1.02–1.08) (21). One study published in 2019 revealed that being exposed to socioeconomic adversity added to the possibility of childhood abuse, which, in turn, was associated with worse maternal and child outcomes (22). In addition, a study published in 2021 examining the relationship between family finances at the age of 11 years and affective symptoms in adulthood found that childhood neglect had a mediating role (23). Thus, childhood trauma may act as an intermediate process between childhood SES and adulthood depression. While the abovementioned research adds to the picture of how SES and trauma are related to mental health in adulthood, empirical evidence supporting the mediating role of childhood trauma in the relationship between childhood SES and depression pathogenesis is lacking.

In the present study, we recruited first and second year university students, aged > 18 years, who did not receive financial support from their families. We aimed to (i) clarify the effect of childhood SES on adulthood depression and (ii) examine the mediation effect of childhood trauma on the relationship between childhood SES and adulthood depression, using a structural equation model (SEM).

## Methods

### Study Design

This was a cross-sectional study to investigate the relationship among SES, childhood trauma, and depressive symptoms in Chinese undergraduate students.

### Ethical Approval of the Study Protocol

Students provided written informed consent before taking part in our survey. Each step of the study was followed and approved (31800928) by the Ethics Committee of Southern Medical University (Guangdong, China).

### Participants

First and second year undergraduate students from Southern Medical University were recruited using cluster random sampling. Southern Medical University enrolls students from most provinces in China. A total of 1,905 students (aged 16–22 years) completed self-reported questionnaires.

Exclusion criteria were as follows: (i) age < 18 years, (ii) missed responses for >10% of the total items, and (iii) apparently fake responses (e.g., selecting the same value repeatedly). Utilizing these criteria, 98 surveys were excluded from analyses, thereby leaving 1,807 (94.9%) valid samples. The study cohort comprised 796 males (44.1%) and 1,011 females (55.9%), with ages ranging from 18 to 22 years (mean ± SD = 20.25 ± 1.54). There was no significant difference in depressive symptoms (*t* = −0.80, *p* = 0.429) or scores for childhood trauma (*t* = 1.47, *p* = 0.142) between male and female participants, but childhood SES reported by females (5.02 ± 1.32) was slightly higher than that for males (4.89 ± 1.36, *t* = −2.06, *p* = 0.041).

### Procedure

The survey was conducted by a trained researcher. Participants were asked to complete the questionnaire truthfully. After completing the questionnaire, participants were compensated with course credit.

### Measures

#### Childhood SES

Childhood SES was assessed using the MacArthur Scale of Subjective Social Status—Youth Version (MSSSS-YV) (24). This measure uses the visual aid of “ladders” to assess family socioeconomic placement in society and personal socioeconomic placement from 1 to 10 (the first “rung” represents the bottom, and the 10th rung represents the top). We chose the first item of the MSSSS-YV and documented childhood SES during four stages (before the age of 6 years, elementary school, middle school, and high school). The mean response from these four items was chosen to represent general childhood SES. Studies have revealed the use of ladder ranking to be more powerful for reflecting the relationship between SES and individual mental health than other traditional objective indicators (25–27). The MSSSS-YV has also shown great reliability in Chinese college students (Cronbach's alpha coefficient: 0.79) (28). In the current study, the Cronbach's alpha coefficient was 0.90. Ferreira et al. suggested that participants who choose the bottom four ladder rungs (1–4) of the MSSSS-YV should be considered to be from a low-SES family (29).

#### Childhood Trauma

Childhood trauma was assessed by the Chinese version of the Childhood Trauma Questionnaire-Short Form (CTQ-SF). This measure was developed by Bernstein and has been used widely to measure various types of childhood maltreatment (30, 31). The scale contains 28 items, and each item is scored using a five-point Likert scale ranging from 1 (*never*) to 5 (*always*). The CTQ-SF has five subscales: emotional neglect (EN), emotional abuse (EA), physical neglect (PN), physical abuse (PA), and sexual abuse (SA), with each subscale consisting of five items. The cutoff points for severe trauma in the CTQ-SF subscales are as follows: EN ≥ 15, EA ≥ 13, SA ≥ 8, PA ≥ 10, and PN ≥ 10 (31, 32). The CTQ-SF has shown good reliability and validity among Chinese college students (33, 34), with the Cronbach's alpha coefficient being calculated at 0.86 in the present study.

#### Depressive Symptoms

We used the Chinese version of the Beck Depression Inventory Version II (BDI-II; 21 items) to measure depressive symptoms. BDI-II is one of the most frequently used and standardized scales to assess depressive symptoms over the previous 2 weeks in adults. The BDI-II comprises 21 items rated on a four-point scale ranging from 0 (*no symptoms)* to 3 (*severe symptoms*). Total scores range from 0 to 63, with a higher score indicating more severe depressive symptoms. Individuals with BDI-II scores > 13 are considered to have depressive symptoms. The items of BDI-II are divided into three dimensions: emotional symptoms, cognitive symptoms, and somatic symptoms (35). The BDI-II has shown good reliability and validity among Chinese college students (36, 37). The Cronbach's alpha coefficient in the current study was 0.88.

### Data Analyses

Preliminary data analyses were conducted using SPSS 22.0 (IBM, Armonk, NY, USA). Distributions of SES, childhood trauma, and depressive symptoms were slightly skewed. Parametric tests (e.g., two-sample *t*-test) are robust, even for heavily skewed distributions if the sample size is large (38, 39). In the current study, the sample size was sufficient. Thus, we first used a two-sample *t-*test to examine the effect of sex on SES, childhood trauma, and depressive symptoms. Then, we calculated the prevalence of low childhood SES, depressive symptoms, and each type of childhood trauma. Comparisons of childhood trauma and depressive symptoms between the high-SES and low-SES groups were made using independent sample *t*-tests. A Pearson's correlation was used to reveal the correlation of the three key variables.

An SEM was adopted to test the hypothesis that childhood trauma mediates the relationship between childhood SES and depressive symptoms in adulthood. The SEM was calculated with AMOS 21.0 (IBM) using maximum likelihood estimation. In this model, childhood trauma with five observed variables (EN, EA, PN, PA, and SA) and depressive symptoms with three observed variables (emotional symptoms, cognitive symptoms, and somatic symptoms) were considered “latent” variables, and childhood SES was estimated as an “observed” variable. The standards of goodness-of-fit indices were as follows: normed fit index (NFI) ≥ 0.90, comparative fit index (CFI) ≥ 0.90, goodness of fit index (GFI) ≥ 0.90, Tucker–Lewis index (TLI) ≥ 0.90, and root mean square error of approximation (RMSEA) ≤ 0.08 (40). A bootstrapping procedure was used to test the direct and indirect effects of childhood SES upon depressive symptoms.

## Results

### Descriptive Statistics and Correlations

We found that 27.1% of students (*n* = 490) reported that they had been raised in a low-SES family. Furthermore, 20.8% of students (*n* = 375) had suffered at least one type of trauma in their childhood. Hence, low childhood SES and childhood trauma appeared to be common among our study cohort.

Physical neglect showed the highest prevalence of all types of childhood trauma (14.8%, *n* = 268), followed by emotional neglect (7.0%, *n* = 126). Additionally, 4.1% of participants had experienced sexual abuse while growing up, while 1.8% suffered physical abuse (*n* = 33). Emotional abuse was the least common type of childhood trauma, with only 30 students (1.7%) reporting it. Finally, 13.3% of students (*n* = 240) were screened to have depressive symptoms.

Table 1 shows that undergraduates from low-SES families demonstrated higher scores on the CTQ-SF and BDI-II than those from high-SES families. Specifically, the prevalence of emotional abuse, physical abuse, emotional neglect, and physical neglect was significantly higher in low-SES students. Moreover, affective, cognitive, and somatic symptoms of depression were more severe in low-SES students. Furthermore, participants with at least one type of childhood trauma had more depressive symptoms than those who did not experience at least one type of childhood trauma (*t* = 10.44, *p* < 0.001).

**Table 1:** Differences between high SES and low SES participants on depressive symptoms and childhood trauma.

**Variables**	**SES ≤ 4** **(***n*** = 490)**	**SES > 4** **(***n*** = 1,317)**	***t***	***P***
	**Mean ± SD**	**Mean ± SD**		
BDI	7.57 ± 6.95	6.22 ± 6.29	3.75	<0.001
Affective	6.36 ± 1.64	6.06 ± 1.49	3.52	<0.001
Cognitive	9.80 ± 3.14	9.09 ± 2.66	4.49	<0.001
Somatic	12.41 ± 3.07	12.08 ± 2.99	2.07	0.039
CTQ-SF	22.11 ± 3.99	20.93 ± 3.65	5.74	<0.001
EA	6.94 ± 2.29	6.46 ± 1.79	4.26	<0.001
PA	5.65 ± 1.30	5.48 ± 1.13	2.57	0.010
SA	5.43 ± 1.04	5.36 ± 1.12	1.11	0.269
EN	9.28 ± 3.85	8.33 ± 3.35	4.85	<0.001
PN	7.88 ± 2.58	6.88 ± 2.17	7.63	<0.001

*BDI, Beck Depression Inventory; Affective, affective factor of BDI; Cognitive, cognitive factor of BDI; Somatic, somatic factor of BDI; CTQ, Childhood Trauma Questionnaire; EA, emotional abuse subscale of CTQ; PA, physical abuse subscale of CTQ; SA, sexual abuse subscale of CTQ; EN, emotional neglect subscale of CTQ; PN, physical neglect subscale of CTQ; SES, socioeconomic status (as determined using the MacArthur Scale of Subjective Social Status—Youth Version)*.

Table 2 shows that childhood SES is negatively correlated with both childhood trauma (*r* = −0.15, *p* < 0.001) and depressive symptoms (*r* = −0.10, *p* < 0.001), while a positive correlation between childhood trauma and depressive symptoms (*r* = 0.34, *p* < 0.001) was observed. The five forms of childhood trauma were also found to be negatively correlated with childhood SES and depressive symptoms.

**Table 2:** Correlations among SES, CTQ-SF, the different forms of CTQ-SF, and BDI (*N* = 1,807).

**Variables**	**M ± SD**	**SES**	**CTQ**	**PA**	**EA**	**SA**	**EN**	**PN**
SES	4.96 ± 1.34	–	–	–	–	–	–	–
CTQ	21.25 ± 3.78	−0.15^**^	–	–	–	–	–	–
PA	5.53 ± 1.18	−0.08^**^	0.71^**^	–	–	–	–	–
EA	6.59 ± 1.95	−0.11^**^	0.83^**^	0.48^**^	–	–	–	–
SA	5.38 ± 1.10	−0.05^*^	0.55^**^	0.23^**^	0.23^**^	–	–	–
EN	8.59 ± 3.51	−0.16^**^	0.48^**^	0.31^**^	0.48^**^	0.15^**^	–	–
PN	7.15 ± 2.33	−0.20^**^	0.56^**^	0.25^**^	0.36^**^	0.23^**^	0.50^**^	–
BDI	6.59 ± 6.50	−0.10^**^	0.34^**^	0.18^**^	0.35^**^	0.14^**^	0.33^**^	0.26^**^

*BDI, Beck Depression Inventory; CTQ, Childhood Trauma Questionnaire; PA, physical abuse subscale of CTQ; EA, emotional abuse subscale of CTQ; SA, sexual abuse subscale of CTQ; EN, emotional neglect subscale of CTQ; PN, physical neglect subscale of CTQ; SES, socioeconomic status (as determined using the MacArthur Scale of Subjective Social Status—Youth Version)*.

* *p < 0.05*;

** *p < 0.01*.

### Mediation Analysis

**Figure 1:**
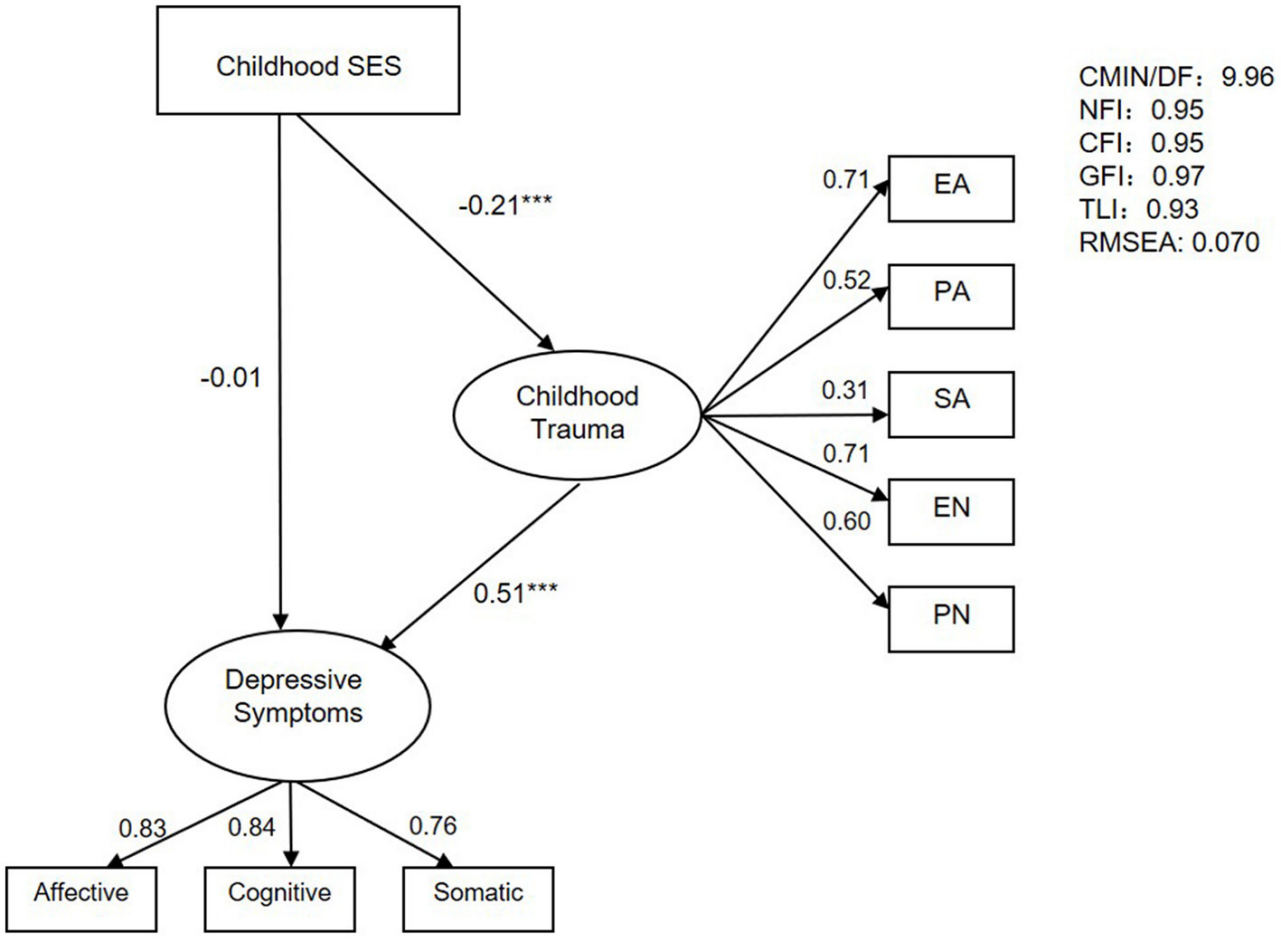
The SEM of the mediating effect of childhood trauma on childhood SES and depressive symptoms. ^***^p < 0.001.

We conducted SEM to test the mediating role of childhood trauma on the relationship between childhood SES and depressive symptoms (Figure 1). The hypothetical model fits the data well (*n* = 1,807, *χ*^2^/df = 9.96, NFI = 0.95, CFI = 0.95, GFI = 0.97, TLI = 0.93, RMSEA = 0.070). The standardized path coefficients from SES to childhood trauma (*β* = −0.21, *p* < 0.001) and from childhood trauma to depressive symptoms (*β* = 0.51, *p* < 0.001) were significant. The direct influence of SES on depressive symptoms was not significant (*β* = −0.012, *p* = 0.607). The total effect in this model was 0.112, and the mediating effect was 0.100, which accounted for 89.3% of the total effect.

Table 3 shows the detailed effect size of direct and indirect pathways in the mediation model. These results indicate that childhood trauma plays a profound mediating role in the link between childhood SES and depressive symptoms in adulthood. Young adults raised in low-SES families tended to experience more trauma during childhood, and more traumatic experiences in childhood may lead to the development of more depressive symptoms in early adulthood.

**Table 3:** The analysis of paths and effects (*N* = 1,807).

**Effect**	**Path**	**Effect size**		
		**Estimated**	**SE**	**95% CI**
Direct	SES → depressive symptoms	−0.012	0.024	(−0.048 to 0.032)
Indirect	SES → childhood trauma → depressive symptoms	−0.100^*^	0.015	(−0.129 to −0.080)
	Total effect	0.112		
	Indirect effect (%, total indirect effect/total effect)	0.100 (89.3%)		

* *p < 0.01*.

## Discussion

First, we investigated associations among childhood SES, childhood trauma, and depressive symptoms in adulthood. Then, we employed an SEM to verify the hypothesis that the effect of childhood SES on depressive symptoms is mediated *via* childhood trauma.

Like previous studies (1–3, 41), the results of the present study demonstrate the link between childhood SES and depressive symptoms in young adults. Young adults from low-SES families exhibited more severe depressive symptoms than those from high-SES families, and depressive symptoms were significantly related to low childhood SES. A recent survey in China that was conducted in a community sample showed that low SES is related to both depressive symptoms and major depressive disorder (3). Therefore, our findings provide further evidence for an important role of childhood SES in the formation of depressive symptoms.

Trauma was a common experience during childhood in the present study: 27.1% of young adults reported at least one type of trauma. Physical neglect was the most prevalent trauma, followed by emotional neglect, sexual abuse, physical abuse, and emotional abuse. Similar to the current findings, a study conducted in the USA showed neglect to be the most prevalent form of child maltreatment (42). In general, we found that childhood trauma was a risk factor for depressive symptoms in young adults. Among all types of childhood trauma, emotional abuse and emotional neglect were the predominant risk factors associated with depressive symptoms (*r* = 0.35 and *r* = 0.33, respectively), followed by physical neglect (*r* = 0.26). Although the prevalence of emotional abuse was 1.7%, the association between emotional abuse and depressive symptoms was the highest. This finding is consistent with a meta-analysis revealing that emotional abuse during childhood showed the highest correlation with depression (43). Thus, childhood emotional abuse is worthy of more attention with respect to depressive symptom prevention. In addition, emotional abuse and emotional neglect showed a modest correlation with each other (*r* = 0.48), and both were closely related to depressive symptoms; therefore, emotional maltreatment, both neglect and abuse, should be granted more attention in research.

As hypothesized, childhood trauma was demonstrated to be associated with childhood SES. Childhood trauma was more likely to occur in low-SES families (Table 2). Physical neglect was the type of trauma with the highest level of relation to SES (*r* = −0.20), followed by emotional neglect (*r* = −0.16), emotional abuse (*r* = −0.11), physical abuse (*r* = −0.08), and sexual abuse (*r* = −0.05). These findings are in accordance with research that found negligence of children to be more common in families with low incomes and in impoverished nations (14, 44).

One study from 2018 suggested that physical abuse and environmental stressors (which include economic stress) that occur during childhood should be considered separately. When these two factors were measured individually and analyzed statistically, the power of predicted prevalence of adult depressive symptoms increased (45). The current study indicates that the role of financial stress and physical abuse in the development of depressive symptoms needs to be distinguished. Matthews et al. summarized that harsh family environments (e.g., domestic conflicts and neglect) are more likely to emerge in low-SES families and that there are psychosocial origins to the connection between SES and health (46). Other studies have also indicated that childhood trauma should be recognized as an important factor contributing to the high incidence of depression in peri-urban settings, which are typically poorer than urban areas (13). The aforementioned studies suggest that the relationship between economic stress and depression may be caused by economically related adverse life events. The present study hopes to provide some new insight to better understand the path from childhood SES to childhood trauma and, finally, to the development of depressive symptoms in later life.

The most important contribution of the current study is to examine the potential mediating effect of childhood trauma on childhood SES and adulthood depressive symptoms using an SEM. For theoretical contributions, we directly evaluated the mediating effect of childhood trauma on the link between childhood SES and adulthood depressive symptoms, and the results confirmed our hypothesis. In this study, the mediating effect accounted for 89.3% of the total effect, indicating that the role of childhood trauma is more important than the direct impact of economic stress on depressive symptoms. A study from 2021 selected two subject groups of different ages, 23 and 50 years old, respectively, and found that childhood neglect played a mediating role between childhood socioeconomic position at 11 years old and current affective symptoms (23). The mediating effect in the 23-year-old population of this study, whose age closely resembled our sample, differed from the current results, at 71.4%. The mediating effect in the present study was relatively high, which may be due to the inclusion of childhood abuse and the use of an SEM. Consistent with the current results, this study also shows that childhood neglect is the core component of mental symptoms caused by economic conditions. Furthermore, the present research also provides a new perspective for the social causation theory. While the social causation theory posits that economic stress leads to depression, a review found that increasing income may be a necessary, but not sufficient, condition for the improvement of health in individuals with low incomes (47). The current research provides a new perspective for the improvement of the social causation theory in that it verifies an important mediating variable between early-life economic stress and later-life depressive symptoms. Our findings also help to emphasize the importance of preventing child maltreatment, in an effort to reduce the prevalence of depressive symptoms observed in low-SES populations in China. In 2017, UNICEF pointed out that China's welfare agencies value financial support more than protection against child abuse (48). The close link between low childhood SES and childhood trauma as well as the profound role of childhood trauma on depressive symptoms observed in the present study, together with the unsatisfactory situation in practice, highlight the need to strengthen child-protection services, while simultaneously providing financial support. While economic adjustment may require long-term effort (49), protection of children can have an immediate impact (50).

### Limitations

Despite the unique contribution to further the understanding of socioeconomic disadvantages, childhood trauma, and their associations with depression, our findings should be interpreted in light of three main limitations. First, we chose the MSSSS-YV to assess subjective SES, without collecting data of the objective financial level, because studies have shown that subjective SES is more closely related to health outcome than objective SES (51). However, objective SES (as indicated by parental employment and family structure) may also contribute to childhood trauma (52). Second, we relied on self-reported measures, which may have attenuated our findings, due to the social desirability effect and recall biases. Third, previous research suggests that current economic status is closely related to depressive symptoms; however, we did not investigate current socioeconomic status. Although the sample of students we recruited in the current study were in a special period, in which they were almost completely financially dependent on the family, is helpful to eliminate the confusion of adult economic status on depression, it also limits the extent of generalizability of the current findings to a wider population.

### Future Direction of Research

The future direction of the current research could aim at refinement of measurement for SES, childhood trauma, and depressive symptoms mentioned here. For instance, assessment of depressive symptoms should be combined with an examiner rating scale and self-rating scale, and a diagnosis from a psychiatrist should be taken into consideration (if necessary). Furthermore, different types of childhood trauma should be subdivided in future research. One study in 2017 showed that suffering multiple traumas was likely to produce various emotional symptoms, so it is necessary to clarify the difference between a single traumatic incident and multiple traumatic incidents in future studies (53). Moreover, researchers should combine prospective and retrospective investigations of childhood trauma to better evaluate the impact of childhood trauma (54).

## Conclusions

We demonstrated the mediating role of childhood trauma in the correlation between low childhood SES and depressive symptoms in young adulthood. Our data supported the social causation theory, which stresses the causal role of low childhood SES for depressive symptom pathogenesis. Our research also contributes to a deeper understanding of the relationship among childhood SES, trauma, and depressive symptoms later in life.

## Data Availability Statement

The raw data supporting the conclusions of this article will be made available by the authors, without undue reservation.

## Ethics Statement

The studies involving human participants were reviewed and approved by the Ethics Committee of Southern Medical University. The ethics committee waived the requirement of written informed consent for participation.

## Author Contributions

XY and YW designed the study and the procedure. PC and YH participated in the survey and collected data. CY and PC analyzed the data and wrote the manuscript. JX participated in revising the manuscript. All authors contributed to the article and approved the submitted version.

## Funding

This work was supported by grants from the National Natural Science Foundation of China (Grant Numbers: 31800928 and 71874075), Humanities and Social Science Fund of Ministry of Education of China (Grant Number: 17YJCZH219), the Degree and Postgraduate Education Reform Project of Guangdong Province (Grant Numbers: 2020JGXM024 and 2021JGXM026), and Guangdong Provincial Philosophy and Social Sciences 13th Five-Year Plan Planning Co-construction Project (Grant Number: GD18XXL04).

## Conflict of Interest

The authors declare that the research was conducted in the absence of any commercial or financial relationships that could be construed as a potential conflict of interest.

## Publisher's Note

All claims expressed in this article are solely those of the authors and do not necessarily represent those of their affiliated organizations, or those of the publisher, the editors and the reviewers. Any product that may be evaluated in this article, or claim that may be made by its manufacturer, is not guaranteed or endorsed by the publisher.
